# 528. Impact of Hormone Therapy on Healing and Complications in Transgender Patients with Previous Burn Injuries

**DOI:** 10.1093/jbcr/irag033.225

**Published:** 2026-04-06

**Authors:** Joshua E Lewis, Olivia Anga, Jarrell Patterson, Derek Nuamah, Chrishaun Alexander, Jordan Kankam, Erin Jackson, Brianna L Gilner, Bryan S Torres, Oyetokunbo Ibidapo-Obe

**Affiliations:** Indiana University Health, Galveston, TX; University of St. Thomas, Houston, TX; Brody School of Medicine East Carolina University, Greenville, NC; Miller School of Medicine, University of Miami, Miami, FL; Morehouse School of Medicine, Atlanta, GA; Texas Tech University Health Sciences Center, Lubbock, Lubbock, TX; Prairie View A&M University, Prairie View, TX; Whiddon College of Medicine at the University of South Alabama, Mobile, AL; Tulane School of Medicine, New Orleans, LA; Memorial Hermann, Houston, TX

## Abstract

**Introduction:**

Transgender and gender-diverse (TGD) individuals experience unique health risks and disparities, yet no research has explored how gender-affirming hormone therapy (HT) may influence adverse burn outcomes. This study aims to evaluate the association between pre-injury hormone therapy (estrogen or testosterone) and adverse complications following burn injuries in TGD patients.

**Methods:**

Using TriNETX database, adult TGD patients who sustained burn injuries and on estrogen or testosterone hormone therapy before their burn-injury were compared to those not on hormone therapy before their burn injury. Outcomes were assessed at three and six months post burn- injury. Outcomes assessed were wound infection, hypertrophic scarring, sepsis, reported pain, and mortality. Cohorts were propensity matched on age, race, ethnicity, burn severity, diabetes, autoimmune and mental health disorders, substance use disorders, and social determinants of health. Multivariable regression models calculated risk ratios (RR) with 95% confidence intervals (CI). Statistical significance was deemed *p*<.05.

**Results:**

At 3 months post-injury, estrogen therapy was associated with significantly higher risks of wound infection (RR = 2.59, *p*=.0126), sepsis (RR = 1.44, *p*=.0047), and reported pain (RR = 1.14, *p*<.0001), and a significantly lower risk of mortality (RR = 0.85, *p*=.0004). Testosterone users showed similar trends with significantly increased risks of wound infection (RR = 1.33, *p*=.0228), sepsis (RR = 1.21, *p*=.0425), and reported pain (RR = 1.10, *p*=.0023), alongside reduced mortality (RR = 0.74, *p*=.0167). At 6 months, estrogen therapy remained associated with higher risks of wound infection (RR = 2.90, *p*=.0078), hypertrophic scarring (RR = 1.83, *p*=.0026), sepsis (RR = 1.61, *p*=.0019), and reported pain (RR = 1.27, *p*<.0001), with further reduction in mortality (RR = 0.47, *p*=.0016). Testosterone therapy similarly increased risks of wound infection (RR = 1.81, *p*=.0172), sepsis (RR = 1.33, *p*=.0054), and pain (RR = 1.09, *p*=.0267), while lowering mortality (RR = 0.59, *p*=.0051).

**Conclusions:**

Pre-injury hormone therapy in transgender patients is associated with increase risks of wound infection, hypertrophic scarring, sepsis, and reported pain and decreased mortality after burn injury. These findings suggest possible effects of hormone therapy that may influence healing and survival, highlighting the need for hormone-aware, personalized burn care protocols. Further studies are needed to elucidate the biological mechanisms underlying these associations and to develop optimized, hormone-informed clinical management strategies for transgender burn patients.

**Applicability of Research to Practice:**

Findings highlight the need for burn care teams to consider hormone therapy in tailoring post-burn care for TGD patients. This work underscores the importance of inclusive research to inform personalized, equitable treatment strategies in burn recovery.

**Funding for the study:**

N/A.

